# Insecticide Resistance Evolution Negatively Affects the Fitness of *Aphis gossypii* Glover During Selection on Cotton Plants Under Laboratory Conditions

**DOI:** 10.3390/plants14162527

**Published:** 2025-08-14

**Authors:** Hina Gul, Ali Güncan, Arzlan Abbas, Zeeshan Ullah, Xie Yuqing, Farman Ullah, Nicolas Desneux, Xiaoxia Liu

**Affiliations:** 1 MARA Key Laboratory of Pest Monitoring and Green Management, Department of Entomology, College of Plant Protection, China Agricultural University, Beijing 100193, China; gulhina680@gmail.com (H.G.); xieyuqingxx@163.com (X.Y.); 2 Department of Plant Protection, Faculty of Agriculture, Ordu University, 52200 Ordu, Türkiye; guncan.ali@gmail.com; 3 School of Agriculture and Food Sustainability, The University of Queensland, Gatton, QLD 4343, Australia; a.abbas@uq.edu.au; 4 Department of Entomology, Abdul Wali Khan University Mardan, Khyber Pakhtunkhwa 23200, Pakistan; zeeshanullah212@gmail.com; 5 Xianghu Laboratory, Institute of Bio-Interaction, Hangzhou 311258, China; farmanullah787@gmail.com; 6 Université Côte d’Azur, INRAE, CNRS, UMR ISA, 06000 Nice, France

**Keywords:** resistance development, fitness costs, demographic parameters, resistance selection, cotton leafroll disease

## Abstract

The cotton aphid, *Aphis gossypii* Glover, is among the most economically significant sap-sucking insect pests, inflicting substantial economic losses worldwide. Insecticides such as thiamethoxam, bifenthrin, and flonicamid are commonly used to manage this pest, despite the inherent risk of developing resistance. In this study, we investigated the evolution of insecticide resistance in *A. gossypii* after continuous selection with thiamethoxam, bifenthrin, and flonicamid over more than ten generations in a controlled laboratory environment. We assessed the fitness of resistant strains using an age-stage, two-sex life table approach, comparing them to a susceptible population. The results indicated that *A. gossypii* achieved resistance levels of 158.60-fold against thiamethoxam, 129.18-fold against bifenthrin, and 104.75-fold against flonicamid. Furthermore, life table analyses revealed that the developmental stages were significantly extended, while longevity decreased in all resistant strains compared to the susceptible population. Additionally, the net reproductive rate (*R*_0_), fecundity, and reproductive days were notably reduced in the resistant cohorts when compared to the susceptible strain. Overall, these findings provide valuable insights into the laboratory-induced evolution of insecticide resistance and the associated fitness costs in *A. gossypii* when feeding on cotton plants. This information could be instrumental in formulating effective resistance management strategies to control this significant pest.

## 1. Introduction

The cotton aphid, *Aphis gossypii* (Glover, 1877) (Hemiptera: Aphididae), is a highly impactful insect pest that feeds on plant sap. It causes substantial economic and environmental damage on a global scale. *Aphis gossypii* inflicts significant agricultural loss through its direct feeding activities, as well as its indirect role in the transmission of viruses, such as cotton leafroll dwarf virus (responsible for cotton leafroll disease) and the excretion of honeydew [1]. Despite several eco-friendly control options [2,3], chemical insecticides are typically the predominant method used to control insect pests, including *A. gossypii*. The indiscriminate use of insecticides can result in sublethal or hormetic effects, promote resistance development, and ultimately hinder effective pest control [4,5,6]. The development of insecticide resistance is widely recognized as a significant contributing factor to the ineffectiveness of chemical-based insect pest control methods, as well as public health concerns [7,8,9]. Additionally, the presence of these invasive species leads to the excessive use of chemical pesticides, which may exhibit hormetic effects on target insect populations while negatively impacting non-target species and posing risks to human health [10,11]. In China, the management of this pest has primarily depended on the use of chemical insecticides. As a result, cotton aphids have developed resistance to a broad spectrum of insecticides, including organophosphates, carbamates, pyrethroids, and neonicotinoids [12,13].

Fitness refers to the ability of an insect population to survive and reproduce successfully in a given environment, relative to other individuals within the same species. The term “fitness cost” refers to the trade-off between multiple traits, where certain alleles that confer increased fitness under insecticide selection pressure result in reduced fitness in other environments, particularly in the absence of insecticides. Insects endure life history trade-offs as a result of resource allocation and physiological constraints. The resistant strains of insect populations experience fitness costs, including alterations to environmental adaptability, leading to changes in their survival and reproductive capacity [14,15]. Furthermore, the presence of fitness costs can hinder the rapid spread of resistance within a population, particularly under certain field conditions [16]. The evolution of insecticide resistance is commonly associated with a significant energy expenditure, resulting in reduced fitness of insecticide-resistant insect populations [17]. Numerous research studies have shown that insecticide resistance in various insect pests is often accompanied by fitness costs, which are significant factors alongside other traits [18,19,20,21,22]. In a study by Ma et al., sulfoxaflor-resistant *A. gossypii* strains exhibited a relative fitness (*R_f_*) of 0.917 compared to susceptible strains [23]. Similarly, sulfoxaflor-resistant green peach aphid, *Myzus persicae* (Sulzer, 1776) (Hemiptera: Aphididae), showed a reduced fitness of 0.83 relative to susceptible individuals [24]. The occurrence of insecticide resistance, along with the associated fitness costs, has been documented in numerous insect pests [22,23,25]. However, there is currently no available information on the evolution of resistance and the associated fitness costs of thiamethoxam, flonicamid, and bifenthrin in *A. gossypii*. The life table is an efficient tool used for assessing the impact of external factors, such as insecticides, temperature, and secondary plant metabolites, on various aspects of insect populations. These aspects include reproductive capabilities, survival rates, growth patterns, fecundity, and life expectancy [26,27]. Conventional life tables were limited in their scope, as they did not account for individual variations and developmental stages. Therefore, incorporating data from different developmental stages in a population allows for more comprehensive investigations using age-stage, two-sex life tables, thereby mitigating the limitations associated with female-based life tables.

In this study, we assessed the potential risk of resistance development in *A. gossypii* when exposed to thiamethoxam, flonicamid, and bifenthrin for ten successive generations. In addition, we compared thiamethoxam, flonicamid, and bifenthrin-resistant strains of aphids to susceptible ones using the age-stage two-sex life table method, which allowed us to precisely measure overall fitness, including fecundity, longevity, and important demographic parameters. The findings on selection-induced resistance and the associated fitness costs of thiamethoxam, flonicamid, and bifenthrin could provide valuable insights into the resistance development in *A. gossypii*. Additionally, these results will offer comprehensive knowledge for the most effective application of insecticides in managing this significant pest.

## 2. Results

### 2.1. Selection-Induced Insecticide Resistance Evolution

*Aphis gossypii* strains resistant to thiamethoxam, flonicamid, and bifenthrin developed after continuous 48 h insecticide exposure over ten generations, with key findings summarized in Table 1, Table 2 and Table 3. The lethal concentrations (LC_50_) values for thiamethoxam, flonicamid, and bifenthrin in susceptible *A. gossypii* were 0.31, 0.37, and 1.82 ppm, respectively. Resistance development in the thiamethoxam-resistant strain started gradually in the first three generations (F1–F3), with LC_50_ values of 0.47, 0.87, and 1.92 ppm, respectively. However, a significant increase in the resistance ratio was observed from the F4 to F10 generation, with LC_50_ values ranging from 3.46 to 49.6 ppm. After ten generations of thiamethoxam selection, *A. gossypii* exhibited a 158.60-fold resistance (Table 1). In contrast, the bifenthrin-induced strain showed a gradual increase in resistance from F1 to F5, with LC_50_ values ranging from 2.52 to 26.2 ppm. Subsequently, a dramatic rise in resistance was observed from generations F6 to F10, resulting in a 129.18-fold resistance against bifenthrin (Table 2). In all cases, flonicamid resistance increased gradually across all generations, with LC_50_ values ranging from 0.41 to 39.0 ppm from F1 to F10, resulting in 104.75-fold resistance (Table 3).

### 2.2. Impact of Insecticide Resistance on Different Developmental Stages of A. gossypii

Life-history traits, including developmental time and longevity among susceptible and resistant strains of *A. gossypii* exposed to bifenthrin, flonicamid, and thiamethoxam, are summarized in Table 4. Thiamethoxam-resistant strains exhibited significantly longer mean developmental durations for the second, third, and fourth instar compared to bifenthrin- and flonicamid-resistant strains, as well as the susceptible strain (*p* < 0.05). However, no significant differences were observed in the development duration of the first instar of *A. gossypii* across strains (*p* > 0.05). Additionally, pre-adult stages of *A. gossypii* were significantly prolonged in thiamethoxam-resistant strains, followed by bifenthrin- and flonicamid-resistant strains, compared to the susceptible strain (*p* < 0.05). In contrast, adult (female) and total longevity were significantly reduced in thiamethoxam-resistant strains, followed by flonicamid and bifenthrin, when compared to the susceptible aphids (*p* < 0.05) (Table 4).

### 2.3. Reproduction and Life Table Parameters of the Insecticide-Resistant Strain of A. gossypii

We presented a comparison of fecundity and life table parameters between susceptible and resistant strains of *A. gossypii* exposed to bifenthrin, flonicamid, and thiamethoxam insecticides in Table 5. The results showed that the net reproductive rate (*R*_0_) was significantly lower in the flonicamid-resistant strain, followed by bifenthrin and thiamethoxam, compared to the susceptible strain (*p* < 0.05). Fecundity (*F*) was significantly lower in the flonicamid- and thiamethoxam-resistant strains, followed by bifenthrin, when compared to the susceptible strain (*p* < 0.05). Additionally, reproductive days (RP*_d_*) were substantially reduced in the thiamethoxam-resistant strain, followed by bifenthrin and flonicamid, relative to the susceptible strain (*p* < 0.05). Furthermore, the intrinsic rate of increase (*r)* and the finite rate of increase (*λ*) were significantly higher in the bifenthrin-resistant strain compared to susceptible strains of *A. gossypii* (*p* < 0.05). The mean generation time was significantly reduced in flonicamid-resistant strains, followed by bifenthrin- and thiamethoxam-resistant strains, compared to susceptible *A. gossypii* (*p* < 0.05). The total pre-reproductive period (TPRP) was significantly longer in thiamethoxam-resistant aphids, followed by bifenthrin and flonicamid strains, compared to the susceptible *A. gossypii* populations (*p* < 0.05). Additionally, the adult pre-reproductive period (APRP) was significantly extended in thiamethoxam- and flonicamid-resistant strains (*p* < 0.05), with no significant differences observed in bifenthrin-resistant strains compared to susceptible *A. gossypii* (*p* > 0.05).

**Figure 1:**
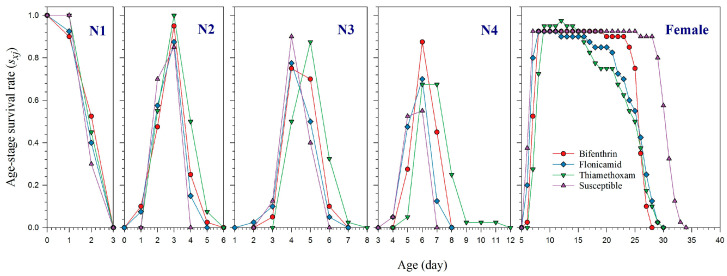
Age-stage specific survival rate (*s_xj_*) of bifenthrin-, flonicamid-, and thiamethoxam-resistant and susceptible strains of *Aphis gossypii*.

**Figure 2:**
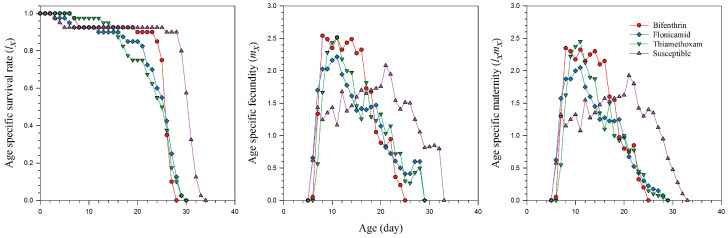
Age-specific survival rate (*l_x_*), age-specific fecundity (*m_x_*), and the age-specific maternity (*l_x_m_x_*) of bifenthrin-, flonicamid-, and thiamethoxam-resistant and susceptible strains of *Aphis gossypii*.

**Figure 3:**
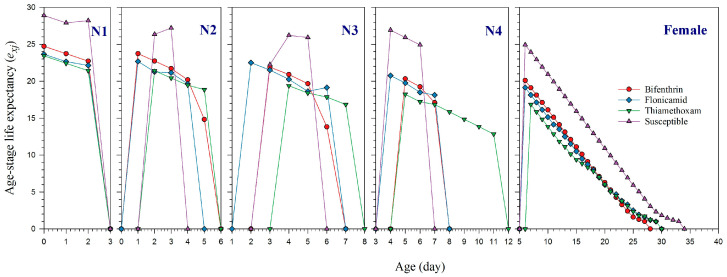
Age-stage life expectancy (*e_xj_*) of bifenthrin-, flonicamid-, and thiamethoxam-resistant and susceptible strains of *Aphis gossypii*.

**Figure 4:**
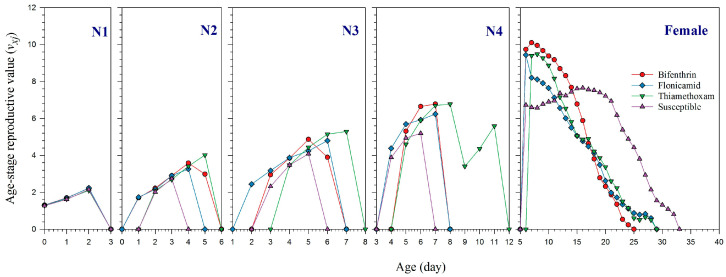
Age-stage reproductive value (*v_xj_*) of bifenthrin-, flonicamid-, and thiamethoxam-resistant and susceptible strains of *Aphis gossypii*.

The *s_xj_* curves reflect the probability of survival for newly born nymphs to reach a specific age *x* and stage *j*. These curves demonstrate that the survival of adult *A. gossypii* strains was significantly affected by resistance to bifenthrin, flonicamid, and thiamethoxam, as compared to the susceptible strain (Figure 1). The *l_x_*, *m_x_*, and *l_x_m_x_* curves show that the lifespan of females resistant to thiamethoxam was 26 days, while those resistant to flonicamid lived for 27 days (Figure 2). Females resistant to bifenthrin had a slightly shorter lifespan of 25 days. In contrast, susceptible females had a longer lifespan, living up to 33 days. The effects of resistance to bifenthrin, flonicamid, and thiamethoxam on the *l_x_*, *m_x_*, and *l_x_m_x_* curves are further highlighted in Figure 2. The *e_xj_* curves illustrate the life expectancy for an individual *A. gossypii* at age *x* and stage *j* to survive beyond age *x*, showing that resistant strains of bifenthrin, flonicamid, and thiamethoxam likely have shorter life expectancy than susceptible aphids (Figure 3). The *v_xj_* curves provide insight into the potential future reproductive output of populations at age *x* and stage *j*. Additionally, the fecundity of *A. gosspyii* notably declined in the resistant strains compared to the susceptible strains (Figure 4).

## 3. Discussion

The application of insecticides remains essential for the global management of aphid populations. However, the evolution of insecticide resistance has diminished the efficacy of these agrochemicals [28,29]. Numerous studies have investigated the development of insecticide resistance in various insect species [23,24,30]. The selection and establishment of insecticide resistance are strongly influenced by potential fitness costs associated with resistance traits [23,31]. To the best of our knowledge, no previous studies have examined the selection-induced resistance to thiamethoxam, bifenthrin, and flonicamid resistance, along with the associated fitness costs, in *A. gossypii*. Therefore, a comprehensive investigation into the potential of resistance evolution and its related fitness trade-offs is crucial for the effective management of *A. gossypii*. Our findings demonstrate that, under laboratory conditions, *A. gossypii* developed high levels of resistance after ten generations of selection, as 158.60-fold to thiamethoxam, 129.18-fold to bifenthrin, and 104.75-fold to flonicamid. Recent studies have reported varying levels of resistance to insecticides in aphid species. For instance, *A. gossypii* has developed 32.64-fold resistance to acetamiprid [22], 23.17-fold to clothianidin [31], and up to 245-fold resistance to sulfoxaflor [23] under laboratory conditions. Similarly, *S. graminum* and *R. padi* have evolved substantial resistance, with 38.57-fold and 34.46-fold resistance to thiamethoxam, 28.67-fold and 31.97-fold resistance to bifenthrin, and 21.31-fold and 26.46-fold flonicamid resistance, respectively [32], following continues selection under laboratory conditions. In other instances, *Spodoptera exigua* (Hübner, 1808) (Lepidoptera: Noctuidae) has exhibited 69.76-fold resistance to deltamethrin and 113.29-fold resistance to gossypol [33]. Likewise, *Bradysia odoriphaga* (Yang & Zhang, 1985) (Diptera: Sciaridae) has developed 43.32-fold resistance to chlorfenapyr and 76-fold resistance to clothianidin [25]. Numerous studies have investigated selection-induced resistance across various pest-insecticide combinations [34,35,36,37,38]. Variability in resistance levels may arise from differences in the origin of field-collected populations, prior exposure to insecticides, and the duration of insecticide-free maintenance under laboratory conditions. If the aphid population continues to be exposed to thiamethoxam, bifenthrin, and flonicamid over additional generations, there is a potential for further increases in resistance ratios. Additionally, different insect species respond variably to insecticides. Extensive research has identified primary mechanisms of insecticide resistance in insects, including metabolic detoxification, target-site mutations, reduced cuticular penetration or increased excretion, and behavioral avoidance. The upregulation of genes involved in these resistance mechanisms is often associated with fitness costs. The evolution of insecticide resistance frequently imposes fitness trade-offs in insects [17,29,38]. Several studies have reported resistance-associated fitness cost in various insects [25,39,40]. In the present study, we specifically evaluated resistance-related fitness costs using four isogenic strains of *A. gossypii*, all derived from the same population.

Our results revealed that the developmental durations of the second, third, and fourth instar nymphs in thiamethoxam-resistant strains were significantly longer compared to both bifenthrin- and flonicamid-resistant strains, as well as the susceptible strain of *A. gossypii*. In contrast, no significant differences were observed in the developmental time of the first instar across all resistant and susceptible strains. Additionally, the total pre-adult developmental period was significantly extended in the thiamethoxam-resistant strain, followed by bifenthrin- and flonicamid-resistant strains, in comparison to the susceptible strain. Our study corroborates previous findings that the pre-adult stages of *S. graminum* were significantly shorter in susceptible strains compared to all-resistant strains [39], while in *R. padi,* the pre-adult stages were significantly prolonged in the thiamethoxam-resistant strain, followed by bifenthrin-resistant strain, compared to the flonicamid-resistant and susceptible strains [32]. Moreover, the developmental and pre-adult durations were substantially prolonged in imidacloprid-resistant *A. gossypii* [41]. Similar trends were observed in *Nilaparvata lugens* (Stål, 1854) (Hemiptera: Delphacidae) and *A. gossypii* strains resistant to nitenpyram and thiamethoxam, which also exhibited significant extensions of developmental stages [21,42]. Furthermore, resistance to insecticides, including imidacloprid, indoxacarb, and deltamethrin, displayed prolonged pre-adult periods in strains of *Musca domestica* (Linnaeus, 1758) (Diptera: Muscidae), *Heliothis virescens* (Fabricius, 1781) (Lepidoptera: Noctuidae), and *Helicoverpa armigera* (Hübner, 1808) (Lepidoptera: Noctuidae), respectively [14,43,44]. These results indicate that prolonged developmental stages represent a key fitness cost associated with insecticide resistance in aphids. This suggests that, in the absence of insecticide selection pressure, resistant strains of *A. gossypii* exposed to thiamethoxam, bifenthrin, and flonicamid are unlikely to proliferate rapidly in field conditions. In contrast, several studies have reported a reduction in the duration of pre-adult stages in certain insect pests [22,23,31]. Adult longevity, fecundity, and reproductive days were substantially reduced in thiamethoxam-, bifenthrin-, and flonicamid-resistant strains compared to the susceptible strain. These findings are consistent with our previous research, which demonstrated that susceptible *S. graminum* and *R. padi* strains exhibited longer longevity, higher fecundity, and reproductive rate. The same pattern was observed in *A. gossypii*, *M. persicae*, and *S. exigua* [22,24,33,42].

A trade-off between specific life-history often arises when both are energetically costly [44,45,46,47]. Under insecticide-induced stress, organisms must allocate limited energy and resources toward detoxification and survival mechanisms [17]. In our study, resistant strains of *A. gossypii* exposed to thiamethoxam, bifenthrin, and flonicamid exhibited prolonged nymphal development, reduced longevity, and decreased fecundity compared to the susceptible strain. These patterns suggest a trade-off in energy allocation between adaptation (i.e., resistance mechanisms) and key life-history traits, in accordance with life-history theory.

The extended duration of immature stages in resistant strains likely increases metabolic costs, delaying the onset of reproduction and reducing the number of generations achievable within a season. While the most apparent fitness costs were seen in adult reproductive performance, the developmental delays also contribute significantly to reduced population growth potential. This indirect impact is critical for understanding long-term population dynamics, even if not all parameters showed differences.

Fitness costs linked to insecticide resistance have been well-documented across numerous insect species [17,39], and our findings support this growing body of evidence. Importantly, the measurable costs observed under selection by three insecticides with distinct modes of action reinforce the notion of a consistent trade-off in energetically demanding life-history traits. These results not only highlight the potential of *A. gossypii* to evolve resistance to commonly used insecticides but also emphasize the associated biological costs, providing valuable insights for resistance management strategies.

## 4. Conclusions

In conclusion, our study highlights the rapid evolution of insecticide resistance in *A. gossypii*, with significant resistance levels emerging from continuous selection under laboratory conditions. The observed fitness costs in resistant strains emphasize the importance of integrated pest management strategies that consider both resistance mechanisms and biological trade-offs. These insights will be crucial for developing effective approaches to mitigate resistance and ensure sustainable control of this economically important pest.

## 5. Materials and Methods

### 5.1. Insects and Insecticide

The *A. gossypii* population used in this study was originally collected from cotton fields in the Xinjiang Uygur Autonomous Region of China in 1999. The current research involved two isogenic laboratory strains. The susceptible strain (SS), an anholocyclic clone, was derived from a single female taken from the original field samples collected in an area where insecticides had not been used. The resistant strains of thiamethoxam (TRS), flonicamid (FRS), and bifenthrin (BRS) were developed from the same population through continuous selection. This process involved gradually increasing the concentration of the respective insecticides based on the LC_50_ values obtained from the bioassay of each preceding generation. The selection process was carried out over ten generations under laboratory conditions. Approximately 5000 adults were screened using the leaf-dipping method with a maintained mortality rate of 60–80%. Both susceptible and resistant strains were reared on cotton seedlings, *Gossypium hirsutum* L. (*Malvaceae*), under controlled environmental conditions of 22 ± 1 °C, 70 ± 10% relative humidity, and a 16:8 h light—dark photoperiod. The thiamethoxam (30%), bifenthrin (10%), and flonicamid (20%) insecticides were purchased from Jiangsu Sword Agrochemicals, Yancheng, Jiangsu, China; Qingdao Haolite Biological Pesticide Co., Ltd. Qingdao, Shandong, China; Qingdao Dongsheng Pharmaceutical Co., Ltd. Qingdao, Shandong, China, respectively.

### 5.2. Toxicity Bioassays

Thiamethoxam, bifenthrin, and flonicamid were diluted to a range of concentrations to evaluate their dose–response toxicity against *A. gossypii*. These concentrations were prepared by serial dilution from the respective stock solutions. Thiamethoxam and flonicamid were diluted to the following concentrations: 2, 1, 0.5, 0.25, 0.125, and 0.625 mg L^−1^. For bifenthrin, the concentrations used were 8, 4, 2, 1, 0.5, and 0.25 mg L^−1^. Cotton leaves were cut into circular discs with a diameter of 20 mm using a sharpened steel punch for use in the bioassays. The leaf discs were immersed in the designated insecticide concentrations or distilled water (control) for 15 s. After treatment, the discs were placed on disposable polyethylene gloves and allowed to air dry. Once dried, the discs were placed abaxial side up (adaxial side down) on the agar beds in 12-well cell culture plates. Agar beds were prepared by boiling a 1% agar solution, pouring it into plates to solidify, and used in bioassays following the commonly applied IRAC guideline [31]. Apterous adult aphids of the same age were then carefully transferred onto the discs and covered with paper (Xuan paper) to ensure containment. Bioassays were performed under a controlled laboratory environment at a temperature of 22 ± 1 °C and a photoperiod of 16:8 h (light—dark). Each insecticide concentration was tested in triplicate, with each replicate consisting of 30 apterous adult aphids. Mortality was assessed 48 h after treatment. Aphids were considered dead if they showed no movement when gently prodded with a soft hairbrush. The LC_50_ values were calculated using probit analysis with POLO Plus 2.0 statistical software (LeOra Software Inc., Berkeley, CA, USA).

### 5.3. Establishing the Resistant Strain

The resistant strains of *A. gossypii* to thiamethoxam, flonicamid, and bifenthrin were derived from a susceptible population through continuous selection over more than ten generations. The acute toxicity of these insecticides was assessed for each successive generation of *A. gossypii* as discussed above. During the selection experiment, the concentrations of all insecticides were gradually increased based on the LC_50_ values obtained from the bioassays of the parent aphids. In each generation, approximately 5000 to 6000 adult aphids were screened, and a consistent selection pressure, resulting in 60–80% mortality, was maintained. The resistance ratio (RR) for each generation was calculated by dividing the LC_50_ values of resistant strains by those of the susceptible strain [31]. Insecticide-free cotton plants were used to maintain the susceptible strain of *A. gossypii*. All strains were kept isolated under controlled laboratory conditions (22 ± 1 °C, 70 ± 10% relative humidity, 16:8 h light—dark photoperiod).

### 5.4. Fitness Comparisons

The fitness of thiamethoxam, flonicamid, and bifenthrin-resistant strains of *A. gossypii* was compared to the susceptible strain using the age-stage, two-sex life table method. Approximately 300 apterous adult aphids per strain were introduced onto newly grown cotton plants for 24 h. Subsequently, 40 newborn nymphs (≤24 h old) from each strain were individually transferred onto untreated 20 mm diameter leaf discs for further assessment. These leaf discs were then inverted and placed onto agar beds containing 1.5 mL of 2% agar, which were positioned in the wells of 12-well cell-culture plates. To prevent escape, the plates were covered with Chinese art paper. Each aphid was considered as a separate replicate for all strains of *A. gossypii*. Daily examination of nymphs from both susceptible and resistant strains was made to collect data on fecundity, longevity, developmental time, and mortality. During the reproductive stages, newly born nymphs were counted and subsequently removed daily. All experiments were conducted according to established laboratory protocols under controlled conditions (22 ± 1 °C, 70 ± 10% relative humidity, 16:8 h light—dark photoperiod).

### 5.5. Life Table Data Analysis

The age-stage, two-sex life table method [48,49] was used to analyze the life table data of both susceptible and resistant strains of *A. gossypii* exposed to insecticides [27,50]. Demographic traits, including intrinsic rate of increase (*r*), finite rate of increase (λ), mean generation time (*T*), and net reproductive rate (*R*_0_), and life-history fitness traits, were determined using the TWOSEX-MS Chart program [50]. To compute variances and standard errors 100,000 bootstrap replicates were employed. Paired bootstrap test was then conducted based on the confidence interval of differences to compare all parameters of both susceptible and resistant cohorts of *A. gossypii* at a 5% significance level [51].

The *l_x_* and *m_x_* were determined using Equations (1) and (2):

(1) $$l_{x}=\sum_{j=1}^{\beta }s_{xj}$$

(2) $$m_{x}=\frac{\sum_{j=1}^{\beta }s_{xj}f_{xj}}{\sum_{j=1}^{k}s_{xj}}$$

where *s_xj_* represents the possibility that a newly born nymph will survive to age *x* and stage *j*. *β* shows number of stages, while *f_xj_* represents age-stage specific fecundity of the individual at age *x* and stage *j.*

The RP*_d_* shows days of reproduction and was estimated using Equation (3):

(3) $$RP_{d}=\frac{\sum_{x=1}^{N_{f}}D_{x}}{N_{f}}$$

where *N_f_* represents number of female adults and *D_x_* shows number of days that a female produced offspring [52].

The *r* shows population growth rate when the time reach infinity, and the population achieve stable age-stage distribution. The insect’s population might be increased at a rate per unit of time. The *r* was calculated using the interactive bisection method and corrected with the Euler–Lotka equation with age indexed from 0 [53]:

(4) $$\sum_{x=0}^{\infty }e^{-r\left(x+1\right)}l_{x}m_{x}=1$$

The *λ* represents population growth rate when time reaches infinity and population attain stable age stage distribution. The population size will increase at the rate of λ per time unit. The *λ* was estimated using Equation (5):

(5) $$\lambda =e^{r}$$

The *R*_0_ indicates the total number of nymphs laid by a single female till death. The *R*_0_ was calculated using Equation (6):

(6) $$R_{0}=\sum_{x=0}^{\infty }l_{x}m_{x}$$

The *T* represents the time required for a population to increase to *R*_0_-fold its current size at a stable rate of increase. The *T* was calculated using Equation (7):

(7) $$T=\frac{\ln R_{0}}{r}$$

The *e_xj_* shows the predicted duration that an individual of age *x* and stage *j* will survive. The *e_xj_* was calculated according to Chi and Su [54] using Equation (8):

(8) $$e_{xj}=\sum_{i=x}^{\infty }\sum_{y=j}^{\beta }{s^{\prime }}_{iy}$$

where *s′_iy_* indicates the probability that an individual aphid of age *x* and stage *j* will survive to age *i* and stage *y* by assuming s′ = 1.

*v_xj_* shows the dedication to future offspring at age *x* and stage *j*. The *v_xj_* was calculated using Equation (9) according to [55]

(9) $$v_{xj}=\frac{e^{r\left(x+1\right)}}{s_{xj}}\sum_{i=x}^{\infty }e^{-r\left(i+1\right)}\sum_{y=j}^{\beta }{s^{\prime }}_{iy}f_{iy}$$

## Figures and Tables

**Table 1:** Selection of *Aphis gossypii* for resistance to thiamethoxam under laboratory conditions.

Gen.	LC_50_ (95%CI) ^a^ mg L^−1^	Slope ± SE ^b^	*χ* ^2^	*p*-Value	RR ^c^
F0	0.31 (0.26–0.37)	2.223 ± 0.206	9.416	0.895	-
F1	0.47 (0.38–0.59)	1.674 ± 0.171	4.678	0.997	1.51
F2	0.87 (0.72–1.10)	1.898 ± 0.186	10.136	0.859	2.77
F3	1.92 (1.52–2.56)	1.637 ± 0.190	4.588	0.997	6.12
F4	3.46 (2.83–4.38)	1.893 ± 0.190	10.477	0.841	11.05
F5	6.65 (5.54–8.14)	2.217 ± 0.260	7.315	0.967	21.24
F6	12.0 (9.29–17.1)	1.766 ± 0.230	4.935	0.996	38.32
F7	20.6 (17.5–24.3)	2.732 ± 0.293	5.110	0.995	65.97
F8	29.0 (24.3–35.3)	2.363 ± 0.282	4.204	0.999	92.67
F9	38.1 (32.0–45.9)	2.120 ± 0.194	13.017	0.672	121.60
F10	49.6 (42.5–58.1)	2.461 ± 0.212	6.542	0.981	158.60

Exposed larvae in bioassay, including control = 420; df = 16. ^a^ 95% confidence intervals. ^b^ Standard error. ^c^ RR, resistance ratio, analyzed as (LC_50_ of resistant strain/LC_50_ of susceptible strain).

**Table 2:** Selection of *Aphis gossypii* for resistance to bifenthrin under laboratory conditions.

Gen.	LC_50_ (95%CI) ^a^ mg L^−1^	Slope ± SE ^b^	*χ* ^2^	*p*-Value	RR ^c^
F0	1.82 (1.52–2.20)	1.951 ± 0.178	13.303	0.650	-
F1	2.52 (2.08–3.12)	1.857 ± 0.178	4.686	0.997	1.38
F2	5.23 (3.90–7.81)	1.387 ± 0.170	3.994	0.999	2.87
F3	9.59 (7.83–12.4)	2.079 ± 0.228	8.421	0.935	5.28
F4	15.5 (11.1–22.5)	2.027 ± 0.271	7.623	0.959	8.51
F5	26.2 (20.7–36.8)	2.074 ± 0.308	5.11	0.995	14.44
F6	49.3 (38.5–69.0)	1.836 ± 0.223	7.163	0.970	27.13
F7	87.7 (70.1–117.8)	1.987 ± 0.250	5.475	0.993	48.28
F8	131 (112–154)	3.104 ± 0.413	12.974	0.675	72.37
F9	183 (151–237)	2.457 ± 0.384	6.629	0.980	100.56
F10	235 (194–293)	2.052 ± 0.207	7.195	0.969	129.18

Exposed larvae in bioassay, including control = 420; df = 16. ^a^ 95% confidence intervals. ^b^ Standard error. ^c^ RR, resistance ratio, analyzed as (LC_50_ of resistant strain/LC_50_ of susceptible strain).

**Table 3:** Selection of *Aphis gossypii* for resistance to flonicamid under laboratory conditions.

Gen.	LC_50_ (95%CI) ^a^ mg L^−1^	Slope ± SE ^b^	*χ* ^2^	*p*-Value	RR ^c^
F0	0.37 (0.32–0.44)	2.403 ± 0.205	8.826	0.920	-
F1	0.41 (0.34–0.48)	2.202 ± 0.189	6.296	0.985	1.09
F2	0.52 (0.43–0.63)	1.945 ± 0.177	4.282	0.998	1.38
F3	0.73 (0.60–0.92)	1.732 ± 0.175	6.038	0.988	1.96
F4	1.30 (1.02–1.81)	1.717 ± 0.218	6.329	0.984	3.50
F5	3.85 (3.21–4.73)	2.312 ± 0.271	9.019	0.913	10.33
F6	7.08 (5.40–10.65)	1.861 ± 0.283	9.92	0.871	19.03
F7	11.97 (9.18–17.26)	1.653 ± 0.202	7.741	0.956	32.19
F8	19.9 (16.6–25.1)	2.391 ± 0.312	4.635	0.997	53.62
F9	29.3 (25.7–33.3)	4.183 ± 0.514	5.071	0.995	78.88
F10	39.0 (33.3–46.7)	3.050 ± 0.406	5.873	0.989	104.75

Exposed larvae in bioassay, including control = 420; df = 16. ^a^ 95% confidence intervals. ^b^ Standard error. ^c^ RR, resistance ratio, analyzed as (LC_50_ of resistant strain/LC_50_ of susceptible strain).

**Table 4:** Duration (days) of different developmental stages (Mean ± SE) in flonicamid, thiamethoxam, and bifenthrin resistant strains and the susceptible strain of *Aphis gossypii*.

Stage		Susceptible		Bifenthrin		Flonicamid		Thiamethoxam
	*n*	Mean ± SE	*n*	Mean ± SE	*n*	Mean ± SE	*n*	Mean ± SE
First-instar	40	2.30 ± 0.07 a	40	2.43 ± 0.11 a	40	2.33 ± 0.10 a	40	2.45 ± 0.08 a
Second-instar	39	1.56 ± 0.08 b	40	1.80 ± 0.11 b	39	1.69 ± 0.09 b	40	2.13 ± 0.06 a
Third-instar	37	1.49 ± 0.08 b	39	1.62 ± 0.09 ab	38	1.50 ± 0.10 ab	40	1.73 ± 0.07 a
Fourth-instar	37	1.22 ± 0.07 c	37	1.68 ± 0.09 a	37	1.43 ± 0.08 b	39	1.74 ± 0.09 a
Pre-adult	37	6.59 ± 0.08 d	37	7.41 ± 0.09 b	37	6.92 ± 0.10 c	39	8.05 ± 0.15 a
Adult	37	24.32 ± 0.22 a	37	18.70 ± 0.21 b	37	18.22 ± 0.63 b	39	15.79 ± 0.74 c
Total longevity	37	30.92 ± 0.25 a	37	26.11 ± 0.23 b	37	25.14 ± 0.61 bc	39	23.85 ± 0.72 c

Standard errors were estimated by using the bootstrap technique with 100,000 resampling. Difference was compared using the paired bootstrap test (*p* < 0.05). The means within a row followed by different lowercase letters indicate significant differences among the treatments.

**Table 5:** Reproduction and life table parameters (Mean ± SE) of flonicamid, thiamethoxam, and bifenthrin resistant strains and susceptible strain of *Aphis gossypii*.

Parameters ^a^	Susceptible	Bifenthrin	Flonicamid	Thiamethoxam
	Mean ± SE	Mean ± SE	Mean ± SE	Mean ± SE
*R*_0_	33.03 ± 1.55 a	28.50 ± 1.35 b	24.55 ± 1.48 c	24.78 ± 1.43 bc
*r*	0.2403 ± 0.0050 c	0.2663 ± 0.0047 a	0.2624 ± 0.0065 ab	0.2475 ± 0.0056 bc
*λ*	1.2716 ± 0.0063 c	1.3052 ± 0.0062 a	1.3000 ± 0.0085 ab	1.2808 ± 0.0071 bc
*T*	14.55 ± 0.18 a	12.58 ± 0.11 bc	12.20 ± 0.21 c	12.97 ± 0.22 b
*F*	35.70 ± 0.47 a	30.81 ± 0.44 b	26.54 ± 1.06 c	25.41 ± 1.32 c
RP*_d_*	22.57 ± 0.27 a	13.73 ± 0.25 b	12.84 ± 0.54 bc	11.36 ± 0.53 c
APRP	0.14 ± 0.06 b	0.05 ± 0.04 b	0.38 ± 0.10 a	0.41 ± 0.09 a
TPRP	6.73 ± 0.11 c	7.46 ± 0.10 b	7.30 ± 0.17 b	8.46 ± 0.17 a

Standard errors were estimated by using the bootstrap technique with 100,000 resampling. Difference was compared using the paired bootstrap test (*p* < 0.05). The means within a row followed by different lowercase letters indicate significant differences among the treatments. ^a^ *R*_0_ = net reproductive rate; *r =* intrinsic rate of increase; *λ* = finite rate of increase; *T* = mean generation time; *F*= fecundity; RP*_d_* = reproductive days; APRP = adult prereproductive period; TPRP = total prereproductive period.

## Data Availability

All data analyzed during this study are included in this published article.
